# Phosphatidylethanol (PEth) in Blood as a Marker of Unhealthy Alcohol Use: A Systematic Review with Novel Molecular Insights

**DOI:** 10.3390/ijms241512175

**Published:** 2023-07-29

**Authors:** Matteo Perilli, Federico Toselli, Lisa Franceschetto, Alessandro Cinquetti, Arianna Ceretta, Giovanni Cecchetto, Guido Viel

**Affiliations:** Legal Medicine and Toxicology, Department of Cardiac, Thoracic, Vascular Sciences and Public Health, University of Padova, Via G. Falloppio 50, 35121 Padova, Italy; matteo.perilli@studenti.unipd.it (M.P.); federico.toselli@studenti.unipd.it (F.T.); lisa.franceschetto@studenti.unipd.it (L.F.); alessandro.cinquetti@studenti.unipd.it (A.C.); arianna.ceretta.2@studenti.unipd.it (A.C.); giovanni.cecchetto.1@unipd.it (G.C.)

**Keywords:** phosphatidylethanol (PEth), unhealthy drinking, AUDIT, LC-MS, molecular mechanisms

## Abstract

The Alcohol Use Disorders Identification Test (AUDIT) and its short form, the AUDIT-C, the main clinical instruments used to identify unhealthy drinking behaviors, are influenced by memory bias and under-reporting. In recent years, phosphatidylethanol (PEth) in blood has emerged as a marker of unhealthy alcohol use. This systematic review aims to investigate the molecular characteristics of PEth and summarize the last ten years of published literature and its use compared to structured questionnaires. A systematic search was performed, adhering to PRISMA guidelines, through “MeSH” and “free-text” protocols in the databases PubMed, SCOPUS, and Web of Science. The inclusion criteria were as follows: PEth was used for detecting unhealthy alcohol consumption in the general population and quantified in blood through liquid chromatography coupled to mass spectrometry, with full texts in the English language. Quality assessment was performed using the JBI critical appraisal checklist. Twelve papers were included (0.79% of total retrieved records), comprising nine cross-sectional studies and three cohort studies. All studies stratified alcohol exposure and quantified PEth 16:0/18:1 through liquid chromatography coupled to mass spectrometry (LC-MS) in liquid blood or dried blood spots (DBS) with lower limits of quantitation (LLOQ) ranging from 1.7 ng/mL to 20 ng/mL. A correlation between blood PEth level and the amount of alcohol ingested in the previous two weeks was generally observed. PEth interpretative cut-offs varied greatly among the included records, ranging from 4.2 ng/mL to 250 ng/mL, with sensitivity and specificity in the ranges of 58–100% and 64–100%, respectively. Although the biomarker seems promising, further research elucidating the variability in PEth formation and degradation, as well as the molecular mechanisms behind that variability, are necessary.

## 1. Introduction

Excessive alcohol consumption has been identified as an important risk factor for illness, disability, and mortality, with an increased prevalence of secondary cardiovascular, liver, cancer, and neurological disorders [1,2]. The World Health Organization (WHO) defines a standard drink as 10 g of pure ethanol and harmful consumption as exceeding two standard drinks per day for both men and women [3], although this threshold has not yet been adopted worldwide [4]. Despite the lack of agreement on definitions, it is globally known that the risk of developing alcohol-related diseases is directly proportional to the amount of alcohol consumed [5]. For this reason, it is crucial for all clinicians to gain information on the patient’s risk of developing an alcohol use disorder or secondary diseases, as well as for forensic experts to have a suitable tool to assess alcohol consumption for its possible legal ramifications.

Clinicians often rely on questionnaires such as Alcohol Use Disorders Identification Test (AUDIT), its short form, the AUDIT-C [6,7], or the “Timeline Followback” (TLFB). These questionnaires are, however, influenced by memory bias and under-reporting [8]. Therefore, biomarkers of alcohol intake are highly useful to identify recent or chronic alcohol consumption and to detect unhealthy drinking patterns [9,10]. Very recent alcohol ingestion can be verified by breath (BrAC), by blood alcohol concentration (BAC), or direct alcohol biomarkers in blood and urine, such as ethyl glucuronide (EtG) and ethyl sulfate (EtS) [9].

Indirect biomarkers reflect the toxic effects of ethanol on organs, tissues, or body biochemistry, such as liver enzymes, carbohydrate deficient transferrin (CDT), mean corpuscular volume (MCV), or gamma-glutamyl-transpeptidase (GGT). These have been traditionally used to identify heavy drinkers or alcohol dependent subjects, but they lack sensitivity for the detection of moderate alcohol consumption. Therefore, in the last ten years, the use of phosphatidylethanol (PEth) has been proposed for identifying persons with hazardous drinking habits, such as binge drinkers, and/or persons with moderate drinking habits (i.e., exceeding 20 g of pure ethanol per day).

PEth represents a group of anomalous negatively–charged diacyl phospholipids formed in different human cells in the presence of ethanol. These lipids are derived in vivo from phosphatidylcholines via transphosphatidylation reaction catalyzed by phospholipase D only in the presence of ethanol [11,12]. PEth formation occurs in different cells and tissues, such as erythrocytes, platelets, lymphocytes, brain, and liver, but Peth lipids accumulate only in red blood cells due to the inactivity of phospholipase C [13]. PEth production is related to some of the pathophysiological effects of ethanol in cells. For example, modulation of cell proliferation through p42/44 and mitogen-activated protein kinase (MAPK) pathways has been observed in hepatocytes in vitro [14], as has an increase in biomembrane fluidity, vesicular fusion, and altered activity of several transporters and enzymes (i.e., Na^+^/K^+^—ATPase, Protein Kinase C, cytosolic phospholipase A_2_) [15]. Since PEth molecules carry two fatty acid chains, potentially differing in length and/or degree of unsaturation, there are several possibilities for PEth variants or molecular species [16]. Chain length is mainly between C14 and C20, while C16:0 and C18:1 are the most common substituents present. Forty-eight (48) different isoforms of blood PEth have been identified [17]. Compared to other biomarkers, PEth quantification can detect even low levels of alcohol consumption over a longer time window, since they are detectable for about three to four weeks of daily uptake of 50 g ethanol and up to approximately two weeks after ceasing alcohol intake [11,18,19]. No gender and/or age-related differences have been observed in relation to PEth concentrations [20,21]. The most common analytical technique employed to quantify PEth in blood is liquid chromatography coupled to mass spectrometry (LC-MS or LC-MS/MS), due to its high sensitivity and ability to distinguish between different PEth molecular species [22,23]. 

The homologues PEth 16:0/18:1 and PEth 16:0/18:2 are those most abundant in human blood [24], usually being quantified for clinical and forensic purposes. Recently, studies on moderate alcohol intake have shown that PEth 16:0/18:1 analysis can help discriminate between abstinence and light/harmless drinking from moderate/unhealthy drinking because of correlation between consumption and PEth levels.

The present systematic review aims at investigating the molecular characteristics of PEth. We also summarize the last ten years of published literature on the use of PEth compared to structured questionnaires, such as AUDIT and AUDIT-C, for identifying subjects with potentially unhealthy alcohol consumption.

## 2. Materials and Methods

This systematic review was carried out following the criteria included in the 2020 Preferred Reporting Items for Systematic Reviews and Meta-Analyses (PRISMA) guide [25]. This study was registered in the “International Prospective Register of Systematic Reviews” (PROSPERO) in 2022 (CRD42022355489), and the detailed prespecified protocol is available upon request.

In August 2022, one author (LF) performed a systematic literature search via “MeSH” and “free-text” protocols in the PubMed, SCOPUS, and Web of Science databases, with time limits 1 January 2011–1 January 2023. Search terms used for PubMed and Web of Science were as follows: (“phosphatidylethanol” [Supplementary Concept] OR Peth OR phosphatidylethanol) AND (forensic OR legal OR biomarker OR marker OR alcohol abuse OR abstinence OR monitoring). A modified string was used for Scopus: “ALL ((phosphatidylethanol OR peth OR phosphatidylethanol) AND (forensic OR legal OR biomarker OR marker OR alcohol AND abuse OR abstinence OR monitoring)).” Subsequently, three authors (LF, AC, and MP) selected papers based on titles and abstracts according to the following inclusion and exclusion criteria.

Inclusion criteria:

A. Titles and abstracts available in the English language.
B. PEth used for detecting unhealthy alcohol consumption in the general population.
C. PEth quantified in liquid human blood or dried blood spots through liquid chromatography coupled to mass spectrometry.
D. Full-text available in the English language.

Exclusion criteria:

E. Opinion papers, editorials, and narrative reviews without novel data.
F. Papers with data only on specific populations (e.g., pregnant women, HIV-positive individuals, etc.).
G. Papers containing only data on the development and validation of analytical methods.
H. Papers on postmortem or autopsy cases.

Not meeting at least one of the inclusion criteria A–D or, conversely, meeting one or more of the exclusion criteria E–H was reason for papers’ exclusion. In cases of doubtful classification based on title and abstract, the full text was retrieved. Any discrepancy in the paper selection was addressed through collegial discussion among four authors (LF, AC, MP, and GV).

Data extraction from the selected articles was performed independently by four authors (LF, MP, AC, and FT), and two of them (AC and MP) included these in a table. In order to ascertain the correctness of the process and minimize subjective judgment, one author (GV) checked the accuracy of the entire data extraction process. The following items were collected from each study: authors, journal, year, features of the study (type of study, subjects involved, main aim, clinical setting, and inclusion and exclusion criteria), characteristics of the investigated population (numbers of subjects and race/ethnicity), methods for estimating alcohol use, analytical method used for PEth analysis, type of measured PEth and concentration, type of collected sample, other biomarkers used, and main results obtained (sensitivity, specificity, positive predictive value, and negative predictive value). Any discrepancies in the data extraction process were settled by consensus discussion performed by five authors (LF, AC, MP, GC, and GV).

A validity assessment of each included manuscript was performed using the JBI critical appraisal checklist for analytical cross sectional-studies or for cohort studies [26], based on type of study. Cross sectional-studies were evaluated on eight quality items, while cohort studies were evaluated on eleven quality items. For each quality item, we indicated with “Yes”, “No”, or “Unclear” to indicate the cases in which the data were properly reported, not reported, or not properly reported, respectively. Finally, “Not applicable” refers to those items that are inconsistent with the study in question.

## 3. Results and Discussion

**Figure 1:**
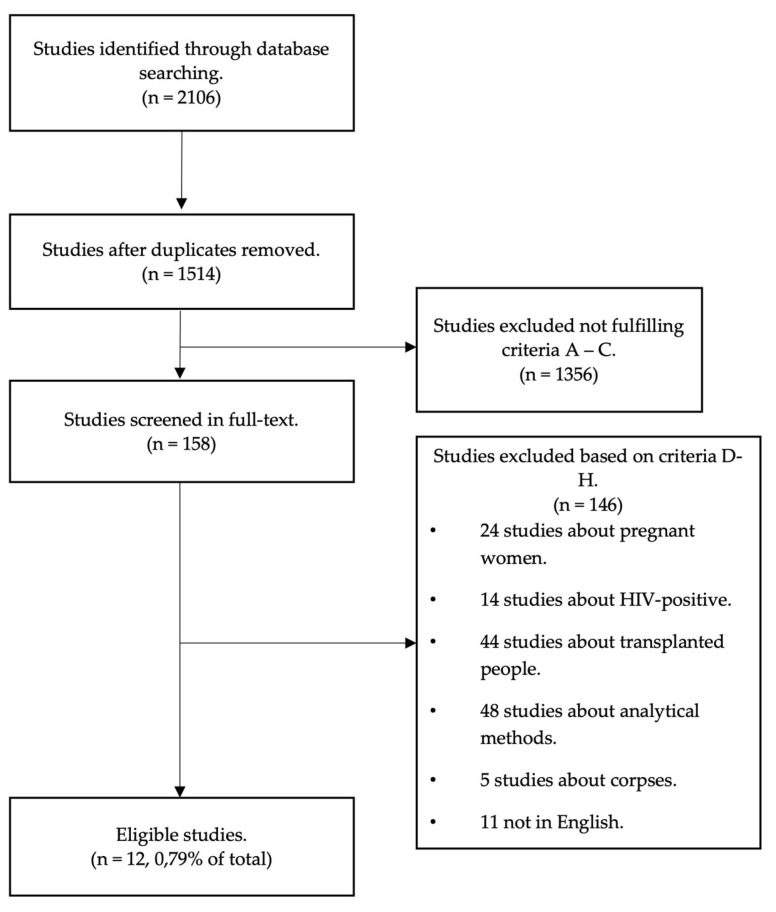
PRISMA flow-chart.

As reported in the PRISMA flow-chart (Figure 1), the combined search on the databases PubMed, Web of Science, and Scopus retrieved 2106 records. Of these, 592 were duplicates and thus were removed, resulting in a total 1514 articles evaluated by title and abstract. From the latter, 1356 were excluded because they did not meet criteria A, B, and C. Of the remaining 158 papers, analyzed in full text, 146 were excluded based on criteria D-H. Twelve papers (0.79% of the total) were included in the present review.

The data extracted from the twelve included papers are presented in detail in the Table S1 of the supplementary material, while the main data are presented in Table 1.

All the included papers were original articles, of which there were nine cross-sectional studies [9,27,30,31,32,33,34,36,37] and three cohort studies [28,29,35] (Table 1). 

**Figure 2:**
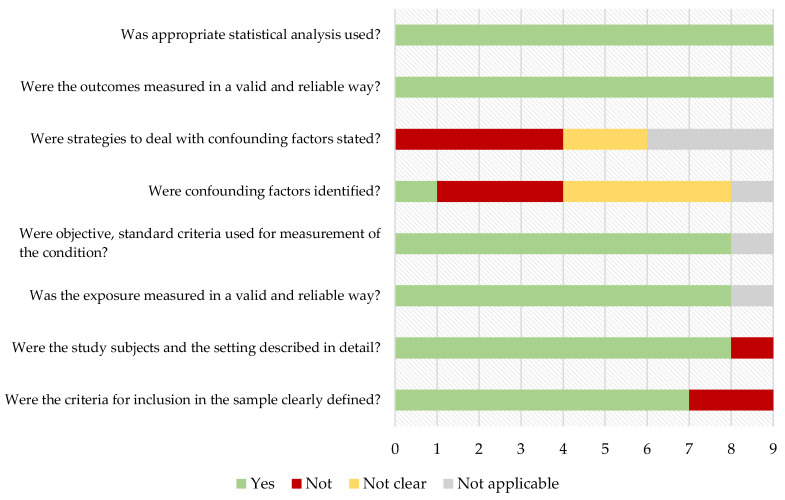
Cross-sectional studies.

**Figure 3:**
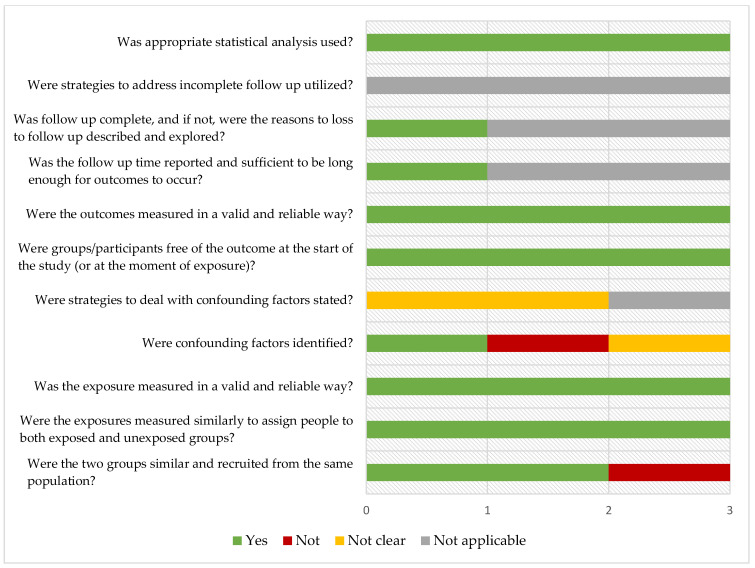
Cohort studies.

Quality assessment results using the JBI critical appraisal checklist for analytical cross sectional-studies or for cohort studies [25] of the included records are displayed in Figure 2 and Figure 3.

In recent years, increasing emphasis has been placed on the detection and treatment of hazardous and harmful drinking disorders, particularly among patients who are seen in primary care settings [38]. 

Hazardous drinking is generally defined as a quantity or pattern of alcohol consumption that places patients at risk for adverse health events, while harmful drinking is defined as alcohol consumption that results in adverse events (e.g., physical or psychological harm). Both hazardous and harmful drinking behaviors are considered “unhealthy” alcohol consumptions [38,39,40].

As defined by the National Institute on Alcohol Abuse and Alcoholism (NIAAA), for women, low-risk drinking is no more than three drinks on any single day and no more than seven drinks per week. For men, it is defined as no more than four drinks on any single day and no more than 14 drinks per week [41]. 

The Alcohol Use Disorders Identification Test (AUDIT) is currently the only clinical instrument specifically designed to identify hazardous and harmful drinking. It allows the investigation of a patient’s alcohol habit through 10 items, returning a numerical value from 0 to 40, with sensitivity of about 90% and specificity of about 80% of detecting an alcohol use disorder [40]. The main limitations of the AUDIT, i.e., the length and time required (about 2–3 min), are partially overcome by the AUDIT-C, which investigates only three items with sensitivity and specificity values of 70% and 90%, respectively [42].

In the included records, AUDIT has been used alone or in combination with AUDIT-C in five records [28,29,30,31,32], AUDIT-C alone in two [9,33], and AUDIT-QF in one [34]. The remaining records were experimental drinking studies [30], or reconstructed previous alcohol exposure by self-reported alcohol intake [27] or by the alcohol intake questionnaire (AIQ) [37]. Lowery et al., who had to reconstruct a potential alcohol misuse among organ donors from proxy history, used the Uniform Donor Risk Assessment Interview [36].

When risk stratification was performed by AUDIT, healthy alcohol use was generally defined for women as AUDIT < 5 and for men as AUDIT < 8 [28,29,32]. When AUDIT-C or AUDIT-QF were used, the thresholds were <6 [31,32] or <4 [33,34], respectively, for identifying harmless alcohol use.

In contrast, Aboutara et al. [27] included two groups with moderate alcohol intake, the first one with a weekly alcohol intake exceeding 24 g and the second one with a weekly alcohol intake exceeding 84 g. 

Regarding PEth molecular species, all the included records identified and quantified PEth 16:0/18:1 in blood; the majority analyzed PEth 16:0/18:1 alone [28,29,30,32,33,34,35,36,37], while the minority analyzed PEth 16:0/18:1 in combination with the 16:0/18:2 [9,31]. Aboutara et al. also investigated PEth 16:0/20:4, 18:0/18:1, 18:0/18:2, and 18:1/18:1 molecular species [27]. 

In all of the included records, liquid chromatography coupled to tandem mass spectrometry (LC-MS/MS) has been used as the qualitative and quantitative analytical technique, with lower limits of quantification (LLOQs) for the homologue PEth 16:0/18:1, ranging from 1.7 ng/mL [33] to 20 ng/mL [9], with the majority of records adopting a LLOQ of 8 ng/mL [27,28,32,36,37] or 4 ng/mL [29,35].

In seven records, PEth was determined in whole venous blood [9,31,32,33,34,35,36], and in four records in dried blood spots (DBSs) [27,28,29,30], a type of sampling in which blood drops are blotted and dried on filter paper. In one record, PEth was quantified in both matrices, demonstrating a strong correlation between PEth quantified in whole liquid blood and in DBS (Spearman’s r = 0.899) [37].

Among the included records, however, there was a certain heterogeneity in the analyzed populations and in the thresholds used to define unhealthy alcohol consumption.Nontheless, a correlation between PEth 16:0/18:1 concentration in blood and the amount of alcohol ingested in the previous two or four weeks was generally observed [9,27,28,29,32,33,34,35]. Specifically in Schrok et al. [9], the correlation of PEth 16:0/18:1 with the measures of alcohol consumption led to Spearman correlation coefficients of r > 0.68 (95% CI 0.61–0.74) for AUDIT-C and r > 0.70 (95% CI 0.64–0.76) for self-reported alcohol consumption in the previous two weeks. Similarly, Gerbase et al. [33] found a significant correlation between blood PEth 16:0/18:1 and the AUDIT-C score (r = 0.617 with 95% CI 0.505–0.729; *p* < 0.001). In contrast, Aboutara et al. [27] showed correlation between the blood concentration of all PEth homologues and the claimed ethanol intake. (The Spearman ranks analysis showed a correlation of 0.73 for PEth 16:0/18:1 and 0.70 for PEth 16:0/18:2, the two most abundant molecular species). In Francis et al. [32], the correlation was stronger for male college students (r = 0.65; *p* < 0.001) than for female college students (r = 0.45; *p* < 0.001). The strongest correlation was observed between PEth concentration, and the total drinks consumed in one occasion (r = 0.68; *p* < 0.001).

It is well-known that PEth synthesis is directly proportional to ethanol exposure and increases with increasing blood ethanol concentration (BAC) [43]; however, it has not yet been elucidated from a molecular point of view why the amount of PEth accumulated in different subjects is different, even if the amount of ethanol ingested is the same and consumed over the same time period [44,45]. 

Several authors have suggested that a better understanding of the inter-individual variability of PLD activity [44,46] might explain the PEth variability, but molecular evidence on that point is still missing. Another important factor is the concentration of ethanol at the site of PLD, which is influenced by the absorption of ethyl alcohol (e.g., affected by percent body fat, genetically determined alcohol, acetaldehyde dehydrogenase, stomach content, drinking pattern, and drinking rate) and its elimination [47]. Drinking experiments in which volunteers were given standard doses of alcohol showed that PEth forms soon after alcohol consumption and that ethanol absorption is a key-factor influencing PEth formation [44,47]. Recently, a large individual patient data meta-analysis [48] has shown that body mass index (BMI) negatively influences PEth sensitivity for detecting unhealthy alcohol consumptions; indeed, blood alcohol concentration per standard drink is inversely proportional to body weight [49]. Therefore, the higher the BMI, the lower the BAC, and thus the formation of PEth. 

There is also uncertainty about the role of other potential factors affecting PEth formation or degradation, such as reactive oxygen species (ROS). Ethanol is metabolized through three enzymatic pathways: alcohol dehydrogenase (ADH), catalase, and the microsomal ethanol-oxidizing system (MEOS). The latter is a multienzyme complex with cytochrome P450 (CYP) and its main isoenzyme, CYP2E1, as the principal elements [50]. The activity of CYP2E1 can be significantly induced by chronic alcohol consumption [51]. It has been demonstrated that, during binge drinking episodes, ethanol is predominately metabolized to acetaldehyde via the MEOS [52,53]; this factor would contribute to the formation of ROS and oxidative stress related to alcohol consumption [54]. The process might also take place outside the liver, given the evidence of the presence of extracellular vesicles containing CYP2E1 in blood, especially in those subjects with alcoholic liver disease [55]. This could be particularly interesting when considering the other constituents of MEOS, such as NADPH–cytochrome P450 reductase and phospholipids [56]. 

Unfortunately, there is still a lack of evidence of a direct involvement of MEOS in the synthesis or degradation of PEth in human erythrocyte membranes. However, what is clear is that PEth may serve not only as a biomarker of drinking behaviors but also as a key pathological factor that affects cell function due to the changes provoked in the lipid composition of the cell membranes.

All the studies included in this review concluded that PEth is a very promising marker of unhealthy alcohol use; however, great variability emerged regarding the adopted cut-offs and the subsequent measured sensitivity and specificity of the biomarker (see Table S1—Supplementary Material). There is growing consensus to refer to PEth values below 20 ng/mL (0.028 µM) as being compatible with abstinence or minimal alcohol consumption during the weeks prior to sampling and to PEth values above 210 ng/mL (0.300 µM; the so-called “Swedish cut-off”) as indicative of chronic excessive alcohol use. On the contrary, there is a lack of consensus on the best cut-off for identifying moderate (but unhealthy) drinking. 

In the included records, the interpretative cut-offs varied between 4.2 ng/mL and 250 ng/mL, with the majority of authors proposing a cut-off between 4.2 ng/mL and 67 ng/mL [9,27,28,32,33,35,36,37] with sensitivity and specificity in the range of 58–100% and 64–100%, respectively. This wide range of cut-offs, along with the fact that PEth can persist at a systemic level and be detected in blood up to two weeks after alcohol ingestion, complicates the interpretation of PEth blood concentrations, as well as the differentiation between chronic excessive drinking and binge drinking behaviors.

In seven of the included studies [27,28,29,30,31,35,36], PEth has been used, along with other direct markers of alcohol use (e.g., BAC or uEtG) [27,28,29] and indirect markers, such as transaminases [27,31,36], CDT [27,28,35,36], gGGT [27,31,35,36], and EtG in hair [30]. PEth has always displayed the best area under the receiver operator characteristics curve (AUC-ROC) compared to the other biomarkers; a combined use of PEth and CDT only slightly increased the diagnostic performance [28,35]. 

## 4. Conclusions

Although the marker PEth 16:0/18:1 seems very promising for detecting and classifying unhealthy drinking behavior, further research is necessary to elucidate the variability in PEth formation and degradation, as well as the molecular mechanisms behind that variability. Clearly, cut-off levels should also be further investigated using valid measures of drinking, days since last drink, and other factors potentially influencing the formation and degradation of PEth (i.e., hemoglobin, hematocrit, BMI, drinking pattern and rate, etc.). Optimally, such an investigation of drinking would be in a controlled experimental setting or measured using frequent BrAC, BAC, or wearable biosensors. Combinations of alcohol measures could also be useful. In a clinical setting, where under-reporting of alcohol use is unlikely, AUDIT and self-report of alcohol consumption could be used in combination with PEth with a low cut-off in order to enhance sensitivity and diagnostic accuracy. In a forensic setting, where the subject under examination and is self-reporting, such reports could be unreliable. Other alcohol biomarkers, such as urinary EtG (with the forensic cut-off of 500 ng/mL) and hair EtG (with the 7 pg/mg cut-off for abstinence in a 3 cm long hair sample) might be used in combination with PEth to confirm abstinence or harmless drinking.

## Figures and Tables

**Table 1:** Main data extracted from the selected articles.

Study and Year	Type of Study	Number of Subjects	Clinical Setting	Subjects Stratification	Type of Sample	Form of Measured PEth	Analytical Method LOQ * Cut-Off #	AUC-ROC of PEth	Other Markers
Aboutara et al., 2022 [27]	Cross-sectional	234 patients attending a liver and kidney clinic	Outpatients	By self-reported alcohol intake and by period of time assessed	DBS from EDTA-blood	16:0/18:1 16:0/18:2 16:0/20:4 18:0/18:1 18:0/18:2 18:1/18:1	LC/MS/MS 16:0/18:1: 8.6 ng/mL * 16:0/18:2: 6.0 ng/mL * 16:0/20:4: 7.7 ng/mL * 18:0/18:1: 6.1 ng/mL * 18:0/18:2: 7.5 ng/mL * 18:1/18:1: 6.6 ng/mL * 10 ng/mL #	For PEth cut-off ≥10 ng/mL and a consumption in the last 4 weeks: ≥24 g/week: 16:0/18:1: 0.78 16:0/18:2: 0.76 16:0/20:4: 0.71 18:0/18:1: 0.70 18:0/18:2: 0.70 18:1/18:1: 0.66 ≥ 84 g/week: 16:0/18:1: 0.93 16:0/18:2: 0.89 16:0/20:4: 0.82 18:0/18:1: 0.82 18:0/18:2: 0.82 18:1/18:1: 0.78	uEtG hEtG CDT AST ALT GGT MCV
Afshar et al., 2022 [28]	Prospective clinical	251 patients attending a trauma center	Inpatients	By AUDIT score: “No unhealthy alchol use” if AUDIT < 5 (F) or <8 (M) “Unhealthy alcohol use” if AUDIT ≥ 5 (F) or ≥8 (M)	DBS from EDTA-blood	16:0/18:1	LC/MS/MS 8 ng/mL * 25 ng/mL #	For PEth cut-off 25 ng/mL: 0.93 (CI: 0.92–0.93) In the external validation: 0.83 (CI: 0.72–0.94)	uEtS uEtG CDT GGT BAC
Afshar et al., 2017 [29]	Cohort	122 subjects: From medical and burn ICU (n = 33) From alcohol detoxification unit (n = 51) Healty volunteers (n = 38) (93 M / 29 F)”	Inpatients Outpatients	By AUDIT score: “Any alcohol misuse” if AUDIT ≥5 (F) or ≥8 (M) “Severe alcohol misuse” if AUDIT ≥13 (F) or ≥16 (M)	DBS from whole blood	16:0/18:1	LC/MS/MS 4 ng/mL * 250 ng/mL # 400 ng/mL #	By AUDIT For PEth as a continuous measure for any alcohol misuse: 0.927 (CI: 0.877–0.977) For PEth as a continuous measure for severe alcohol misuse: 0.906 (CI: 0.850–0.962) By AUDIT-C For PEth as a continuous measure for any alcohol misuse: 0.948 (CI: 0.910–0.956) For PEth as a continuous measure for severe alcohol misuse: 0.913 (CI: 0.856–0.971)	BAC
Baggio et al., 2020 [30]	Single-center with a cross-sectional design	233 subjects of army recrutiment centre (233 M/0 F)		By AUDIT score: “Low score” if AUDIT < 13 “High score” if AUDIT ≥ 13	DBS from whole blood	16:0/18:1	LC/MS/MS 90 ng/mL # 210 ng/mL # (excessive chronic drinking)	0.617	hEtG
Cherrier et al., 2020 [31]	Cross-sectional	183 subjects (121 M/62 F)	Outpatients	By age: Middle age subjects (35–59 years) Older age subjects (over 60 years) By AUDIT-C score: “Subjects at-risk for excessive alcohol consumption” if AUDIT-C ≥ 6 “Subjects without risk for excessive alcohol consumption” if AUDIT-C < 6	EDTA-Whole blood	16:0/18:1 16:0/18:2 Total PEth	LC/MS/MS 16:0/18:1: 0.009 μmol/L * 16:0/18:2: 0.03 μmol/L *	-	GGT AST ALT Bilirubin
Francis et al., 2015 [32]	Cross-sectional	202 college students and casual labourers (161 M/41 F)	Outpatients	By AUDIT score: “Low risk drinking” if AUDIT < 8 “Risk drinking” if AUDIT ≥ 8 By AUDIT-C score: “No hazardous drinking” if AUDIT-C < 6 “Hazardous drinking” if AUDIT-C ≥ 6 By TLFB: “Heavy alcohol intake” if were consumed ≥6 S.D. per drinking event	EDTA-Whole blood	16:0/18:1	LC/MS/MS 0.01 μmol/L * 0.01 μmol/L # (any alcohol intake) 0.30 μmol/L # (heavy alcohol intake)	AUDIT ≥ 8 use against PEth for heavy alcohol use: 0.89 (0.83–0.92) AUDIT-C ≥ 6 use against PEth for heavy alcohol use: 0.89 (0.84–0.93)	-
Gerbase et al., 2020 [33]	Prospective cross-sectional	238 adult patients presenting for trauma (161 M/77 F)	ER department of Novo Hamburgo (population: 250,000) in South Brazil	“By AUDIT-C score: “No alcohol misuse” if AUDIT-C < 3 (F) or <4 (M) “Any level of alcohol misuse” if AUDIT-C ≥ 3 (F) or ≥4 (M) “Severe alcohol misuse” if AUDIT-C ≥ 6”	EDTA-Whole blood	16:0/18:1	LC-MS/MS 1.67 ng/mL * 18,3 ng/mL# (any alcohol misuse) 23,9 ng/mL# (severe alcohol misuse)	For PEth cut-off 18.3 ng/mL to detect any alcohol misuse based on AUDIT-C ≥ 3 (F) or ≥4 (M): 0.791 (CI: 0.722–0.860) For PEth cut-off 29.3 ng/mL to detect severe alcohol misuse based on AUDIT-C ≥ 6: 0.885 (CI: 0.830–0.939)	-
Jorgenrud et al., 2021 [34]	Cross-sectional	2874 patients in Oslo: 931 with AUDIT-QF data and PEth levels ≥ 0.030 μM 3009 patients in Moscow: 953 with AUDIT-QF data and PEth levels ≥ 0.030 μM	2 Hospitals in Oslo and Moscow	By AUDIT-QF: “Harmful alcohol use”: ≥5 (M)/≥4 (F) By weekly grams of alcohol: “Harmful alcohol use”: ≥350 g of alcohol	Whole blood	16:0/18:1	UHPLC-MS/MS ≥300 μmol/L # (excessive alcohol use)	For PEth as a continuous variable ≥ 0.030 μM (AUDIT-QF ≥ 5 (men)/4 (women) as cutoff for harmful alcohol use): Oslo: 0.633 (CI: 0.596–0.669) Moscow: 0.685 (CI: 0.651–0.718, *p* < 0.001) For PEth as a continuous variable ≥ 0.030 μM (weekly grams of alcohol ≥ 350 as cutoff for harmful alcohol use): Oslo: 0.856 (CI: 0.798–0.914) Moscow: 0.746 (CI: 0.700–0.793, *p* < 0.001)	-
Kechagias et al., 2015 [35]	Prospective randomized	44 subjects (12 M/32 F)	Department of Clinical Chemistry, University Hospital, Lund, Sweden	Randomization to alcohol abstention or to alcohol consumption: Abstention: avoid any sort of alcohol intake during the three study months Consumption: 300 mL of red wine (32–33 g of alcohol) per 24 h (M); 150 mL of red wine (16–16.5 g of alcohol) per 24 h (F).	Whole blood	16:0/18:1	LC-MS/MS 0.005 μmol/L * (3.5 ng/mL)	For PEth to descriminate between abstention and moderate daily consumption of red wine for 3 months: 0.92 (CI: 0.82–1)	CDT MCV GGT AST ALT
Lowery et al., 2018 [36]	Cross-sectional	140 brain dead organ donors 62% (n = 87) from the Gift of Hope (GOH) donor cohort 38% (n = 53) from the Loyola University Medical Center (LUMC) cohort	Itasca, IL. Loyola University Medical Center (LUMC)	By UNOS definition: “Heavy alcohol use” consumption ≥ 2 S.D./day By CDC definition: “Heavy alcohol use”: >1 S.D. per day on average or ≥4 S.D. consumed on one occasion in one month (F) or >2 S.D. per day on average or ≥5 S.D. consumed on one occasion in one month (M)	Whole blood	16:0/18:1	Online-SPE-LC-MS/MS 8 ng/mL * LOD: 2 ng/mL	For PEth cut-off ≥84 ng/mL to detect alcohol misuse: 0.86 (CI: 0.76–0.94)	AST ALT GGT CDT
Piano et al., 2015 [37]	Cross-sectional	103 subjects (36 M/67 F)	Participants of a larger ongoing study examinating the cardiovascular effects of binge drinking	By Alcohol Intake Questionaire (AIQ) “Alcohol abstainers”: ≤1 S.D. per month in the last 2–3 years (and abstention cannot be due to a medical illness or prior alcohol abuse) “Moderate or social drinkers”: ≤3 S.D. per sitting with ≤1–2 times per week (M); ≤2 S.D. per sitting with ≤1–2 times in a given week in the last 5 years (F). “Binge drinkers”: ≥5 S.D. either on one occasion or within a 2-h period in the last 30 days (M); ≥4 S.D. on one occasion or in a 2-h period in the last 30 days (F); binge drinkers must have had ≥2 binge drinking episodes in the last month.	Venous whole blood Venous DBS	16:0/18:1	HPLC LC/MS/MS Whole blood: 20 ng/mL * >20 ng/mL # (moderate to heavy drinking) DSB: 8 ng/mL * >8 ng/mL # (moderate to heavy drinking)	-	-
Schrock et al., 2017 [9]	Cross-sectional study	300 subjects (203 M/94 F/3 not specified)	Outpatients	By AUDIT-C: Group A “Abstinence” (Group A) if AUDIT-C is 0 Group B “Moderate consumption” (Group B) if AUDIT-C is 1–3 (F) or 1–4 (M) Group C “Excessive consumption” (Group C) if AUDIT-C is ≥4 (F) or ≥5 (M)	Whole blood	16:0/18.1 16:0/18:2	Online-SPE-LC–MS/MS 20 ng/mL * LOD: 10 ng/mL 112 ng/mL # (for PEth 16:0/18:1, to distinguish moderate from excessive consumers) 67 ng/mL # (for PEth 16:0/18:2, to distinguish moderate from excessive consumers)	-	-

PEth: Phosphatidylethanol. S.D.: Standard Drink. AUDIT: Alcohol Use Disorders Identification Test. DBS: dried blood spot. Online-SPE-LC–MS/MS: online solid-phase extraction and liquid chromatography-tandem mass spectrometry. LC MS/MS: Liquid chromatography–mass spectrometry. UHPLC-MS/MS: ultra-high performance liquid chromatography–mass spectrometry. BAC: blood alcohol concentration. uEtG: urinary ethylglucuronid. hEtG: hair ethylglucuronid. CDT: Carbohydrate-deficient transferrin. AST: aspartate aminotransferase. ALT: alanine aminotransferase. GGT: gamma-glutamyl transferase. MCV: mean corpuscular volume. In the 8th column the symbol * identifies the Limit of Quantitation (LOQ) while the symbol # identifies the interpretative cut-off chosen by the authors.

## Data Availability

No new data were created or analyzed in this study. Data sharing is not applicable to this article.

## Supplementary Materials

The following supporting information can be downloaded at: https://www.mdpi.com/article/10.3390/ijms241512175/s1.
